# Using Finite Element Approach for Crashworthiness Assessment of a Polymeric Auxetic Structure Subjected to the Axial Loading

**DOI:** 10.3390/polym12061312

**Published:** 2020-06-09

**Authors:** Ali Farokhi Nejad, Roozbeh Alipour, Mozafar Shokri Rad, Mohd Yazid Yahya, Seyed Saeid Rahimian Koloor, Michal Petrů

**Affiliations:** 1 Faculty of Engineering, School of Mechanical Engineering, University Technology Malaysia, Skudai 81300, Malaysia; yazidyahya@utm.my; 2 Department of Mechanical and Aerospace Engineering, Politecnico di Torino, 10129 Torino, Italy; 3 Department of Mechanical Engineering, Mahshahr Branch, Islamic Azad University, Mahshahr, Iran; r.alipour@mhriau.ac.ir; 4 Department of Mechanical Engineering, Lorestan University, Khorramabad, Lorestan 68151, Iran; shokrirad.m@lu.ac.ir; 5 Institute for Nanomaterials, Advanced Technologies and Innovation (CXI), Technical University of Liberec (TUL), Studentska 2, 461 17 Liberec, Czech Republic; michal.petru@tul.cz; 6 Department of Aerospace Engineering, Faculty of Engineering, Universiti Putra Malaysia, Serdang 43400, Malaysia

**Keywords:** finite element method, crashworthiness, cellular structures, axial loading, negative Poisson’s ratio

## Abstract

Polyurethane foams are one of the most common auxetic structures regarding energy absorption enhancement. This present study evaluates the result reliability of two different numerical approaches, the H-method and the P-method, to obtain the best convergence solution. A polymeric re-entrant cell is created with a beam element and the results of the two different methods are compared. Additionally, the numerical results compare well with the analytical solution. The results show that there is a good agreement between converged FE models and the analytical solution. Regarding the computational cost, the P-method is more efficient for simulating the re-entrant structure subjected to axial loading. During the second part of this study, the re-entrant cell is used for generating a polymeric auxetic cellular tube. The mesh convergence study is performed on the cellular structures using the H- and P- methods. The cellular tube is subjected to tensional and compressive loading, the module of elasticity and Poisson’s ration to calculate different aspect ratios. A nonlinear analysis is performed to compare the dynamic response of a cellular tube versus a solid tube. The crashworthiness indicators are addressed and the results are compared with equivalent solid tubes. The results show that the auxetic cellular tubes have better responses against compressive loading. The primary outcome of this research is to assess a reliable FE approach for re-entrant structures under axial loading.

## 1. Introduction

Modern technology requires new physical material properties. Study of the material using negative Poisson’s ratio (NPR) is taken into account as an option for material property enhancement [1]. Utilizing re-entrant structures with NPR provides the ability to improve mechanical properties in different applications such as medical, automotive, textile engineering etc. [2,3]. Generally, changing the chemical properties and cell shape are two methods for improving mechanical behaviour [4,5]. Most of the researches in this area focus on changing the chemical elements [6,7,8]. Recently, an attempt is being made to enhance the mechanical properties by changing the cell shape of the auxetic structures. Concerning the elastic region, the mechanical properties of materials are mainly influenced by four elastic constants that are elastic moduli or Young’s moduli (E), shear moduli (G), bulk moduli (K) and Poisson’s ratio (ν). Poisson’s ratio is defined as the ratio of transverse contraction strain to longitudinal extension strain concerning the direction of stretching force applied. The formula of Poisson’s ratio contains a minus sign. Therefore, general materials possess a positive Poisson’s ratio [9]. Based on elasticity theorem, the mentioned constants for isotropic materials are dependent on the following set of equations (Wei Yang et al., 2004) [4]:

(1) $$E=\frac{9KG}{\left(3K+G\right)},\;G=\frac{E}{2\left(1+\nu \right)},\;K=\frac{E}{3\left(1-2\nu \right)},\;\nu =\frac{1}{2}\frac{\left(3K-2G\right)}{\left(3K+G\right)}.$$

To enhance the G and K of the structure, considering that E is not a variable, Poisson’s ratio is the option that can be improved to obtain the desired G and K [9,10]. Factually, most of the materials have a positive Poisson’s ratio and the materials with a negative Poisson’s ratio are made by foam and porous materials such as polyurethane or aluminium foams [11,12,13].

Some research has been conducted to convert the material with positive Poisson’s ratios to the NPR [14]. However, the NPR structure can be made by isotropic material in the macro scale, thus, in inter-atomic bonds the re-entrant strucre with NPR can be observed. Several studies have been conducted to examine the global stiffness of auxetic structures. Generally, cellular auxetic structures are designed by repeating a unit cell, and their effective stiffness can be determined through a unit cell [15,16]. To obtain a negative Poisson’s ratio, many different designs such as re-entrant cells as well as rotating rectangles and triangles, arrow-heads, and star-shaped configurations are proposed [17,18,19]. One of the most interesting auxetic structures is the re-entrant cell with scale-independent properties [20,21]. The deformation behaviour of re-entrant structures as a cellular material can be implemented at every scale range from nano scale [22] to a macro level.

The effect of cell-wall alignment on the dynamic responses of a re-entrant honeycomb structure was studied by Zhang et al., [23]. It was recognized that increasing the impact speed, cell angle and relative density leads to increasing the crashworthiness capability of the auxetic structure. A similar investigation on the hexagonal structure has been carried out by Hu and his colleagues [24], where it was realized that a honeycomb with a cell angle of 45° had better energy absorption performance when subjected to impact loading. Three different deformation patterns under impact loading of the cellular structures were discussed by Zou et al., [25]. Based on the collapsing mechanism of hexagonal honeycomb structures, an analytical formulation for the energy absorption ability was derived by Hu et al., [26]. They later developed an analytical model validated by simulations to anticipate the crashworthiness of hexagonal honeycombs under low impact loading [27]. To exhibit their auxetic property, auxetic materials should possess substantial porosity in their microstructures [1]. Therefore, geometric complexity and porosity in these structures under numerical study make their analysis cumbersome. Regarding terms of finite element work, previous studies in this area show that the numerical simulation of energy absorption by auxetic materials, especially in three-dimensional re-entrant structures, is still limited and sparse, thus needing further development [28].

Impact resistance and energy absorption of auxetic structures is also an interesting topic to which researchers have been paying much attention [29,30,31]. Reid and Peng [32] developed the one-dimensional shock theory estimating the characteristics of a crushing front through the wood subjected to uniaxial impact. Ruan et al., [33] performed finite element analysis (FEA) to study the effectiveness of impact speed and the wall thickness of a cell on the localized deformation state and plateau stress. The FE result highly depends on the element size and degree of the polynomial solution. The solution convergence in the FE problem is the most crucial matter for obtaining a single correct solution [34]. Different methods are presented to demonstrate the numerical convergence. The different ways for increasing the model’s degree of freedom can be categorized into two main branches: element refinement (H-method) and increased polynomial degree with the highest accuracy (P-method) [35]. The H-method result will be more accurate by increasing the number of elements. Using a finer mesh, in other words, brings more accuracy to the model. However, choosing the number of fine mesh needs expertise and increases the computational cost as well. The P-method utilizes the complex shape function, keeping constant the number of elements. The initial iteration uses the first-order polynomial shape function and, in the following run, the order of the shape function can be increased [34]. While the difference between the two last iterations remains in a specific tolerance, the simulation is running. Recently, a combination of the H- and P-methods has been used to take advantage of the desired element size as well as the exponential rate of convergence.

Several studies have been carried out on cellular structures in experimental, numerical, and analytical approaches [36,37,38]. However, when the structures are small in scale, especially in micro or nano-scale, the mathematical models are not precise enough to be validated with an experiment. Therefore, a reliable and fast response model is crucial to simulate the dynamic response of the cellular structure. This present study has several aims: (1) To formulate the total strain energy of a 3D re-entrant cell analytically; (2) To evaluate the effectiveness of the different numerical convergence methods for the re-entrant structure subjected to axial loading using the FE model with the beam element generated; (3) Examining the strain energy of the H-version compared to the P-version regarding CPU time and number of elements; (4) Development of a unit cell that generates a cellular tube with NPR behaviour, as well as, (5) The crashworthiness indicators of the proposed structures compared to the conventional tube with different aspect ratios. The FE formulation can be proposed as an efficient and reliable model when a complex cellular structure is subjected to axial loading.

## 2. Analytical Solution for 3D Re-Entrant Cellular Structure

The analytical model used in the present study was developed by Shokri Rad et al., [9], which is based on Castigliano’s theorem. According to this model, for a linear elastic solid, the energy theorem mentioned below presents the relationship among the potential strain energy saved in a beam element, the load, and the bending moment distribution across the same component:

(2) $$\frac{\partial U_{t}}{\partial P}=\delta \;\;\;\;\;,\;\;\;\;\;\;\frac{\partial U_{t}}{\partial M}=\emptyset$$

where:

(3) $$U_{t}=\frac{1}{2}\int \left(\sigma_{xx}\varepsilon_{xx}+\sigma_{yy}\varepsilon_{yy}+\sigma_{zz}\varepsilon_{zz}+\tau_{xy}\gamma_{xy}+\tau_{xz}\gamma_{xz}+\tau_{yz}\gamma_{yz}\right)dV$$

δ is the corresponding deflection by the bending moment magnitude, M. P, is the applied axial force and ∅ is the beam slope provided by the bending moment. *V* is the unit volume of structure and, also, *σ_ii_* and *ε_ii_* are the elements of stress and strain tensors, respectively. The strain energy for a one-dimensional (1D) beam element can be calculated along the length of the beam. Therefore, if a beam is subjected to a bending moment composed by an axial load, then the energy can be calculated as:

(4) $$U_{t}=\frac{1}{2EI}\int {\left(\sigma_{xx}\right)}^{2}dV\;\;=\;\;\int_{0}^{L}\frac{M^{2}}{2EI}+\int_{0}^{L}\frac{P^{2}}{2AE}$$

**Figure 1:**
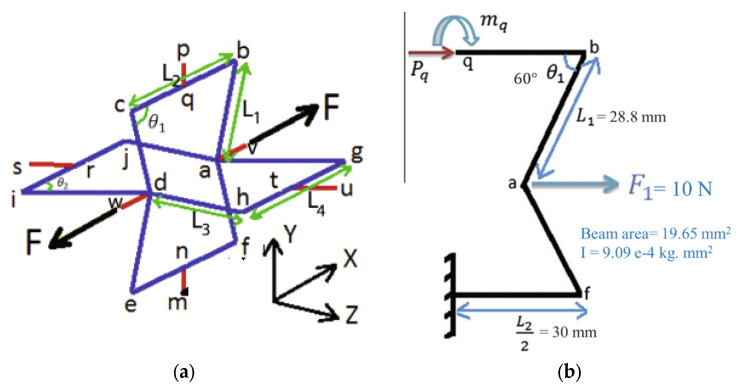
(**a**) the 3D re-entrant cellular structure; (**b**) Real force and moment applied to the simplified model.

Referring to Figure 1a, *m* is located at the centre of the rod connected to the neighbouring cell and the structure is symmetric. Also, it is assumed that the connecting rod, *mn*, is rigid. Hence, the point n is fixed as well. As a result, considering the symmetric property of the cellular structure and point *n* being fixed, Figure 1b can be considered to render the analysis easier. Regarding this figure, $P_{q}$ and $m_{q}$ are the axial force and bending moment at the centre of the beam bc. (point q in Figure 1a), respectively. Modula of elasticity, second moment of inertia and cross section area are denoted by *E, I,* respectively. Table 1 shows the nomenclatures used in this paper.

Due to the symmetric characteristic of the model, it can be assumed that the beam bc has no horizontal deflection and slope at the center (Point q). Therefore, the total strain energy in Figure 1b can be written as:

(5) $$\begin{array}{c} U_{t}=\frac{{m_{q}}^{2}}{EI}[L_{1}+\frac{L_{2}}{2}]+\frac{{P_{q}}^{2}}{AE}[\frac{L_{2}}{2}+L_{1}{cos}^{2}\theta_{1}]+\frac{{P_{q}}^{2}{sin}^{2}\theta_{1}}{EI}[\frac{{{4L}_{1}}^{3}}{3}+{L_{1}}^{2}L_{2}]\\ +\frac{m_{q}{sin\theta }_{1}}{EI}[{2P_{q}L_{1}}^{2}+P_{q}L_{1}L_{2}+\frac{{F_{1}L_{1}}^{2}}{2}+\frac{F_{1}L_{1}L_{2}}{2}]\\ +\frac{{F_{1}}^{2}{sin}^{2}\theta_{1}}{EI}[\frac{{L_{1}}^{3}}{6}+\frac{{L_{1}}^{2}L_{2}}{4}]+\frac{{F_{1}}^{2}}{AE}[\frac{L_{2}}{4}+\frac{L_{1}{cos}^{2}\theta_{1}}{2}]\\ +\frac{F_{1}P_{q}AE}{\left[\frac{L_{2}}{2}-L_{1}{cos\theta }_{1}\right]}+\frac{F_{1}P_{q}{sin}^{2}\theta_{1}}{EI}[\frac{{{5L}_{1}}^{3}}{6}+{L_{1}}^{2}L_{2}]-\frac{{F_{1}}^{2}{L_{1}}^{3}}{2EI}{sin}^{2}\theta_{1} \end{array}$$

To simplify the above equation, we define:

(6) $$\alpha_{1}=\sin\theta_{1}(2{L_{1}}^{2}+L_{1}L_{2})$$

(7) $$\beta_{1}=\left(2L_{1}+L_{2}\right)$$

(8) $$\gamma_{1}=-\left(\frac{\sin\theta_{1}}{2}\right)\left({L_{1}}^{2}+L_{1}L_{2}\right)$$

(9) $$\alpha_{2}=[\frac{\left(L_{2}+2L_{1}{cos}^{2}\theta_{1}\right)}{AE}+\left(\frac{{sin}^{2}\theta_{1}}{EI}\right)\left(\frac{8{L_{1}}^{3}}{3}+2{L_{1}}^{2}L_{2}\right)]$$

(10) $$\beta_{2}=[\left(\frac{\sin\theta_{1}}{EI}\right)\left(2{L_{1}}^{2}+L_{1}L_{2}\right)]$$

(11) $$\gamma_{2}=[-\frac{L_{2}}{2AE}+\frac{L_{1}\cos\theta_{1}}{AE}-\frac{5{L_{1}}^{3}{sin}^{2}\theta_{1}}{6EI}-\frac{{L_{1}}^{2}L_{2}{sin}^{2}\theta_{1}}{EI}]$$

(12) $$\left[\begin{array}{cc} \alpha_{1} & \beta_{1}\\ \alpha_{2} & \beta_{2} \end{array}][\begin{array}{c} P_{q}\\ m_{q} \end{array}]=[\begin{array}{c} \gamma_{1}F_{1}\\ \gamma_{2}F_{2} \end{array}\right]$$

To define the virtual force and moment as a function of applied force it can be written as follows:

(13) $$P_{q}=\frac{\left[\beta_{2}\gamma_{1}-\beta_{1}\gamma_{2}\right]}{\left[\alpha_{1}\beta_{2}-\beta_{1}\alpha_{2}\right]}F_{1},\;m_{q}=\frac{\left[\alpha_{1}\gamma_{2}-\gamma_{1}\alpha_{2}\right]}{\left[\alpha_{1}\beta_{2}-\beta_{1}\alpha_{2}\right]}F_{1}$$

Let us define:

$$\alpha =\frac{\left[\beta_{2}\gamma_{1}-\beta_{1}\gamma_{2}\right]}{\left[\alpha_{1}\beta_{2}-\beta_{1}\alpha_{2}\right]}$$

$$\beta =\frac{\left[\alpha_{1}\gamma_{2}-\gamma_{1}\alpha_{2}\right]}{\left[\alpha_{1}\beta_{2}-\beta_{1}\alpha_{2}\right]}$$

To obtain:

(14) $$P_{q}=\alpha F_{1}\;\;\;\;,\;\;\;\;m_{q}=\beta F_{1}$$

Therefore, by substituting Equation (14) into Equation (5), the total strain energy for a re-entrant cell can be calculated.

## 3. Finite Element Approaches and Simulation

Here, the nonlinear finite element code ABAQUS/Implicit is employed to conduct the computer simulations. The geometry should be presented in micro or nanoscale, but to illustrate the effect of the FE result, the geometry is assumed in macro scale. Both unit cell and re-entrant structures are generated from the beam element (B3). To compare the effect of mesh refinement through the H- and P-versions, the elastic properties of aluminium alloy taken from a previous study [34] are considered for the unit cell and re-entrant structures. These properties are shown in Table 2.

To calculate the structure’s module of elasticity and Poisson’s ratio, the simulation is performed in the elastic region. Both the H- and P-versions’ refinements are employed for simulations. To reduce the time of calculation, and to simplify the FE formulation, the material properties of aluminium are used rather than polymeric material. Actually, the material properties should be taken from a polymeric material, however, to study the overall structural behaviour, an elastic–plastic material like aluminium shows a clear response when a structure is subjected to a crushing load. Here, the region of interest is only the elastic region hence, the overall behaviour of the structure, regardless of material type, can be used as the guideline for using another kind of material such as auxetic polyurethane foam [14]. It can be said that the outcome results of this simulation are far from a real test, however, to perform a qualitative study this procedure is acceptable [1,9,22].

### 3.1. Unit Cell Modelling

Two models with different mesh refinement approaches were used. Regarding the H-version approach, the size of the mesh was refined gradually from 10 to 2 mm to achieve convergence and result stability. The one-dimensional beam element with a linear interpolation function was used in this case. Conversely, the P-version approach used only the one-dimensional element, so the size of the element remained 10 mm, however, the interpolation function was different by using first-, second- and third-order polynomial interpolation functions. The dimensions to generate the FE model were taken from Table 1.

**Figure 2:**
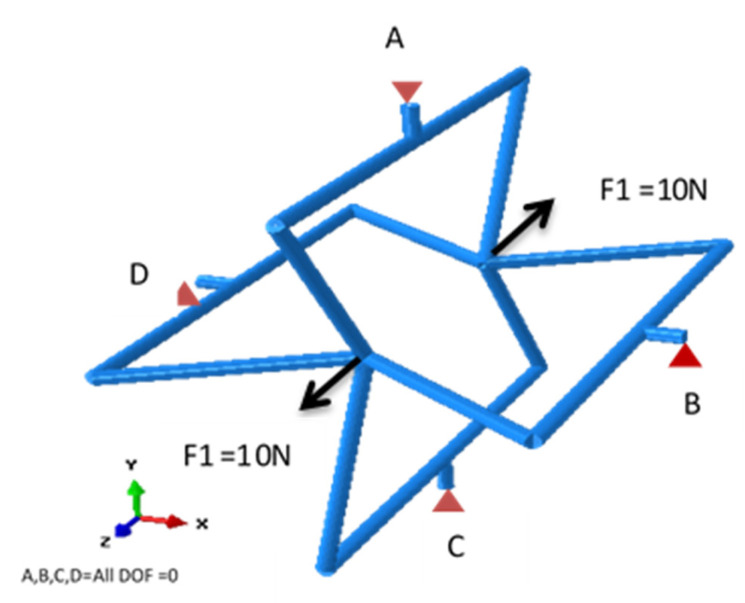
The applied forces and boundary conditions induced on the rendered beam structure.

Figure 2 illustrates the applied loads and boundary conditions assigned to the rendered beam model. A couple of 10 N axial forces were applied at the centre of the structure along the Z direction, and all four neighbouring connecting rods were fixed in all degrees of freedom.

### 3.2. Cellular Tube Modelling

**Figure 3:**
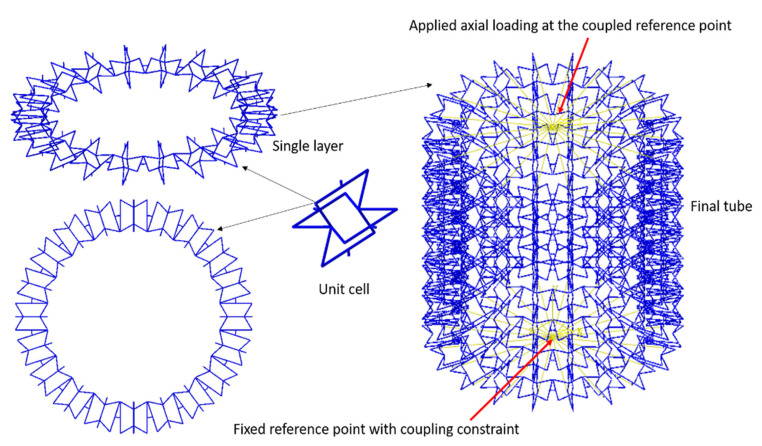
The geometry of a unit cell, single layer, and cellular tube used for FE model.

To generate a polymeric auxetic cellular structure, a unit cell was repeated in a circular direction. Repeating the single layer along the axial direction generated the cellular tube. Figure 3 shows a unit cell, a single layer, and the final geometry of a cellular tube. Dividing different lengths of tubes over the average diameter of the tube (382 mm) made different aspect ratios (L/D). Different aspect ratios (L/D = 1–5) were considered to investigate the mechanical properties of the polymeric auxetic structure subjected to the tension and compressive loading. A reference point was connected to the lower set of nodes at the bottom centre of the cellular tube. The upper set of nodes of the tube were connected to the upper reference point to apply the axial loading. To verify the mesh convergent study, the element size from the unit cell was used for both H-version and P-version methods. Regarding this, the results for 2, 3 and 5 mm element sizes for the H-version were compared with a second-order polynomial function. As a result, the converged values of stresses, strain energy and CPU time were compared.

When a compressive loading is applied to the structure it is possible to have a contact between beam links. To avoid penetration of different beam links together, a general contact algorithm was considered. The general contact method includes surface-to-surface self-contacting between different beams. The tangential behaviour followed the penalty method with a 0.09 friction coefficient, and the normal behaviour was considered as the hard contact algorithm [39]. The main objective of a mesh convergence study for FE simulation is to reach a stable value of stress or magnitude of energy. Therefore, showing strain energy and, especially, von Mises stress are two main criteria that prove the validity of the numerical simulation, whereas, using principal stresses in this study due to the non-linear behaviour of the material and geometry was not appropriate. Hence, the von Mises stress is a key value to justify the FE method results [40,41,42,43,44,45].

**Figure 4:**
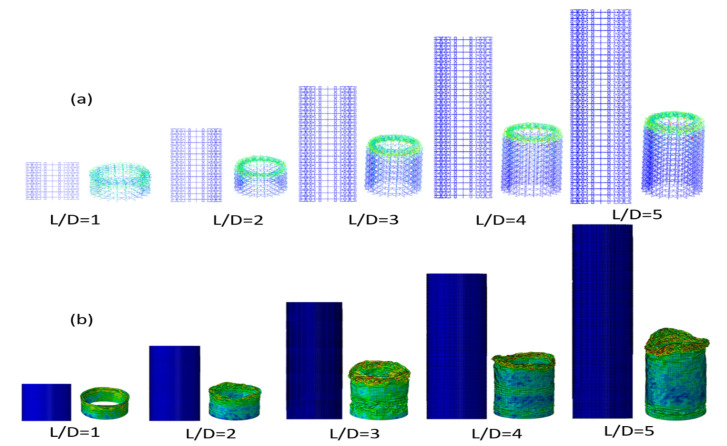
The deformed and unreformed shape of (**a**) cellular tubes and (**b**) conventional tubes with different aspect ratios.

During the next step, to study the energy absorption of the cellular structures with different aspect ratios under compressive loading, a non-linear plastic behaviour was used [34]. Considering each tube, the applied load was increased as ramp until the maximum deflection reached 70% of the initial length of the cellular tube. Occurring there, the energy absorption of the structure from the area under the load-displacement curve was estimated. To compare the impact resistance capability, an equivalent solid tube was designed. Both structures (cellular tubes and solid tubes) had the same average diameter and the same weight. Figure 4 shows the deformed and unreformed shape of cellular tubes and conventional tubes with different aspect ratios.

## 4. Results and Discussion

### 4.1. Unit Cell Modelling Results

#### 4.1.1. H-Method Results for Convergence Study of a Unit Cell

**Figure 5:**
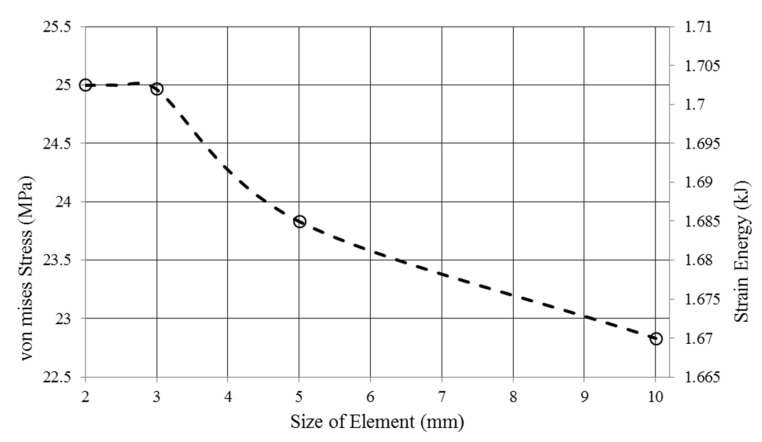
H-method mesh refinement based on corresponding stress and strain energy.

The key point in the FE solution problem is the rate of convergence and, subsequently, the accuracy of the results. Figure 5 demonstrates the mesh convergence analysis using the H-method FE results. It can be seen that the initial course mesh was not appropriate for prediction of the structure behaviour and, by refining the element size, the results became stable and finally remained at a certain amount of strain energy. The converged strain energy and stress at the end of the H-method refinement were 1.7 kJ and 25.02 MPa, respectively.

**Figure 6:**
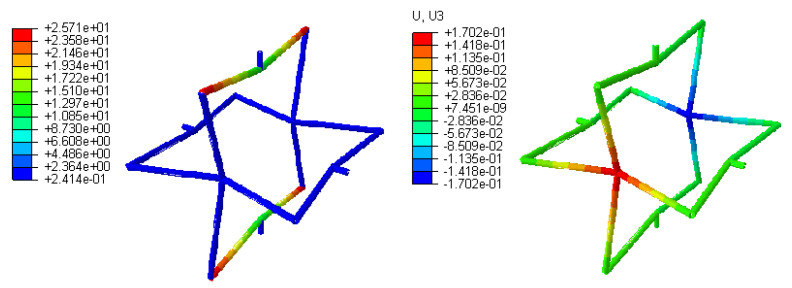
H-method stress distribution and axial deformation after getting a converged solution.

Additionally, Figure 6a,b shows the von Mises stress and overall deformation of the re-entrant cell, respectively, for the H-method analysis. The degrees of freedom for the structure was varying from 300 to 1452, and the CPU time was 0.8 to 1.7 s as well. Decreasing the size of the element from 10 mm to 2 mm made it complex to attain the converged solution. To evaluate a structure with various numbers of cells, the computational cost would be increased significantly.

#### 4.1.2. The P-Method Convergence Study for a Unit Cell

**Figure 7:**
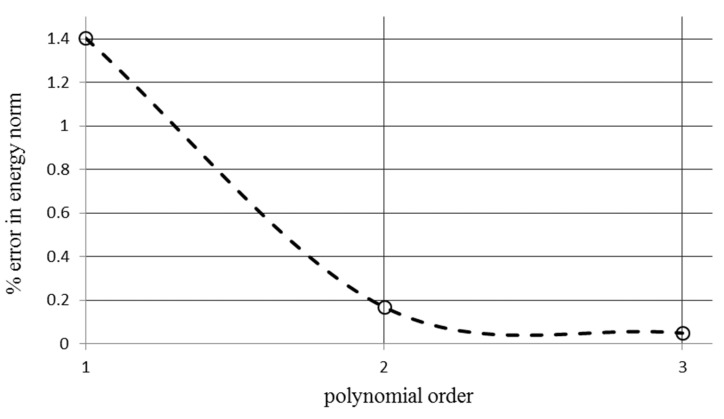
P-method mesh refinement shows reduction of the error of the norm energy by increasing the order of the polynomial function.

Figure 7 depicts the convergence study for the P-method analysis with three different polynomial orders. The second-order polynomial error showed that increasing the higher order polynomial function was not necessary and the second-order polynomial function could be utilized for simulation reliability.

The degree of freedom and CPU time for the second-order polynomial function were 612 and 0.8 s, respectively. Comparison between the P-method and H-method, regarding corresponding stress after convergence, represents a less than 1% error. The stress and deformation contour indicate that the re-entrant cells are capable of absorbing energy. Table 3 compares the result of different simulations between the H-method and P-method.

#### 4.1.3. Unit Cell Strain Energy

**Figure 8:**
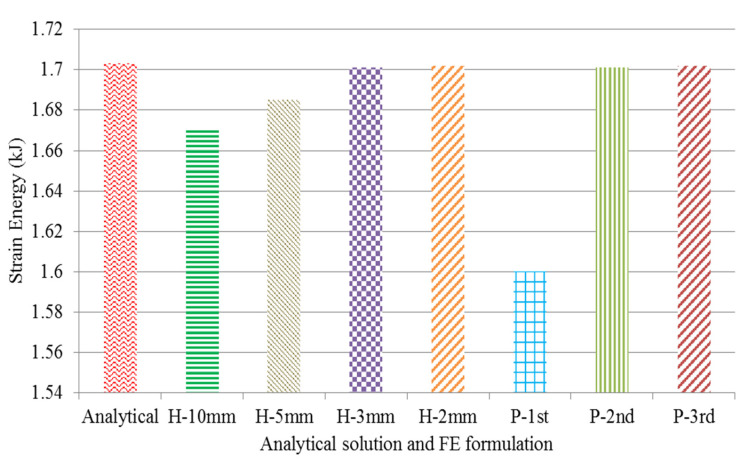
The indication of different numerical results in comparison with the analytical solution.

The total strain energy from equation 5 was calculated and the result can be seen in Figure 8. The magnitude of the strain energy using the analytical method for the re-entrant cell was 1.703 kJ and, in comparison with the converged numerical solution, the error was less than 1%. However, the exact solution can be useful to highlight the effect of convergence in an FE simulation.

### 4.2. Cellular Tube’s Structure Results

#### 4.2.1. Convergence Study for Cellular Tube Structure

Table 4 compares the results of different FE methods for a cellular tube with L/D = 1. The second-order polynomial P-version results presented greater efficiency in terms of strain energy and CPU time. It can be said that using a second-order P-version FE formulation increased the speed of calculation significantly. Comparing the results of the second-order FE formulation with converged H-version results (2 mm element size) shows that this method can accelerate the calculation time 287 s faster.

#### 4.2.2. Structure Stiffness and Poisson’s Ratio

**Figure 9:**
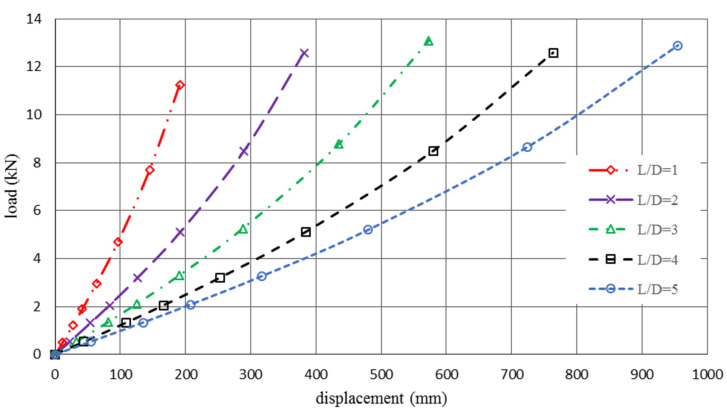
The load-displacement curve of cellular tubes with different aspect ratios.

To calculate the structure elasticity and Poisson’s ration, the cellular tubes with different aspect ratios were subjected to axial loading. The load and displacement data for every 10% loading increment were recorded, and corresponding elastic modules were estimated. The simulation was stopped when the elongation reached 50% of the initial length. Moreover, the Poisson’s ratio of each cellular tube was calculated over time. Figure 9 shows the load-displacement curve of the polymeric auxetic tube under tensile loading with different aspect ratios.

**Figure 10:**
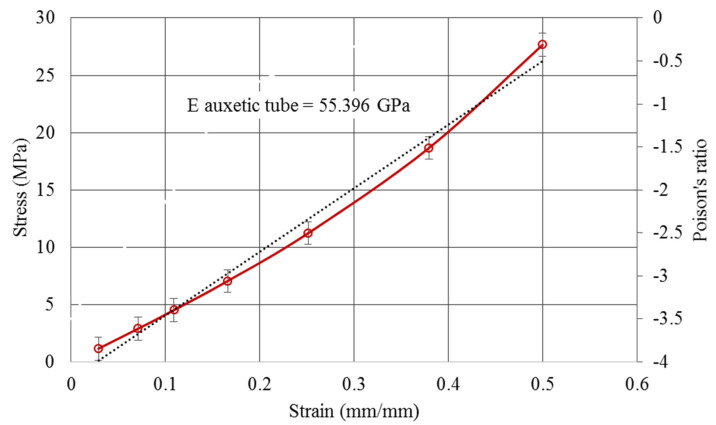
Average structural stiffness and Poisson’s ratio for a cellular tube.

Having transferred the load-displacement to the stress–strain value, the module of elasticity and Poisson’s ratio can be calculated from equation (1). Figure 10 shows the average module of elasticity and corresponding Poisson’s ratio of structure. Considering this figure, it can be interpreted that the Poisson’s ratio can be varied in the elastic region using negative values. After applying 50% of the yield stress on the cellular tubes, the structures still had a negative Poisson’s ratio. The average module of elasticity for all cellular tubes was 55.39 GPa and the Poisson’s ratio of the structure, after 50% elongation, reached −0.5. The error bars in this figure present the upper and lower bounds of different stress magnitudes related to the different aspect ratios of the cellular tubes. The red curve is the mean value of the stress–strain curve of the cellular tube. The black line also represents the slope of the stress–strain curve as the average module of elasticity of all cellular tubes.

Auxetic structures are intrinsically weak under bending and torsion. These kinds of structures are designed for applications which are subjected to axial loading, in other words. This is the reason, proven in several studies [1,2,14], that using auxetic polyurethane foam as a filler foam for structures under compression increases the impact resistance of structures significantly. The value of the shear modulus for the present auxetic structure can be calculated using Equation (1), however, due to the negative Poisson’s ratio of the structure, the shear modulus has a negative or near to zero value. Therefore, in this study the shear modulus calculation is neglected.

### 4.3. Energy Absorption Evaluation

**Figure 11:**
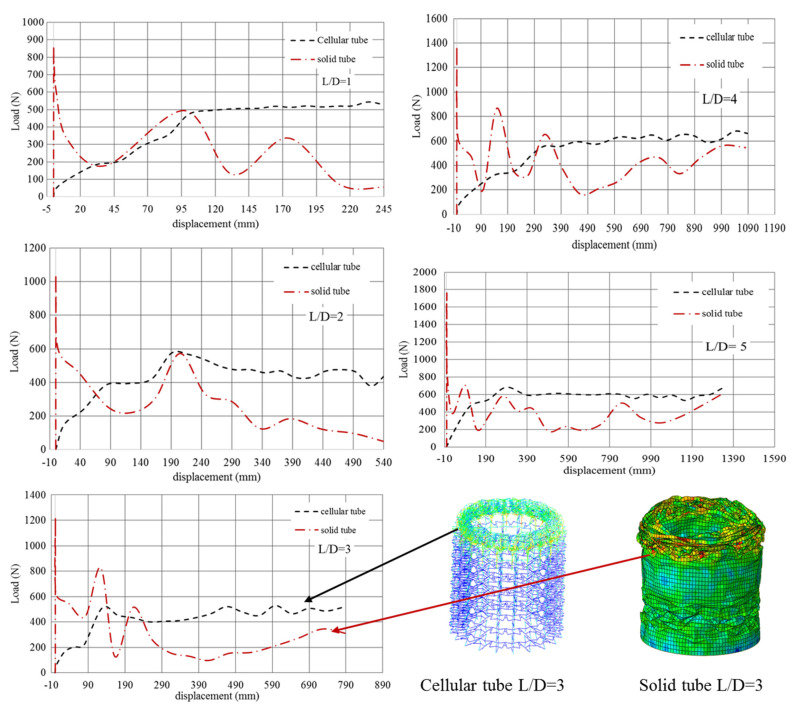
Load-displacement curve for solid and cellular tubes with different aspect ratios.

Figure 11 demonstrates the dynamic responses of cellular tubes and conventional tubes with different aspect ratios. It can be seen that the load-displacement curve from the cellular tube presented a better energy absorption capability. Concerning the conventional tubes, the first peak load was higher than the cellular tube, however, the applied load had a sharp drop over time. Conversely, the loading response from the cellular tube showed that the compressive behaviour of the structure had a good progressive collapse as opposed to the conventional tubes. This phenomenon can be considered as the significant characteristic of the auxetic cellular structure subjected to compressive loading.

Table 5 presents the crashworthiness indicator for conventional and cellular tubes with different aspect ratios. Calculation of the area under load-displacement cure yielded the energy absorption of different samples. The other crashworthiness indicators such as maximum peak load (P_max_), mean crush load (P_ave_), crash force efficiency (CFE) and specific energy absorption (SEA) can be estimated from ref. [5].

Comparing the results of solid tubes and cellular tubes show that using a cellular structure can increase the crash resistance more than 30%. The interesting point regarding the cellular structure is the progressive collapse and increase of the mean crash load in comparison with conventional tubes. To design lightweight components, polymeric auxetic cellular structures could be a good option rather than foam-filled tubes when subjected to axial loading. Comparison of the results of cellular tubes with different L/D shows that increasing the value of the L/D will affect the crashworthiness indicators. Considering all crashworthiness indicators, an increasing trend alongside an increasing L/D can be observed. However, from Figure 10, it can be said that the stiffness of structure for all L/D were almost the same. This value can be seen by comparing the value of *P_max_* from Table 5.

## 5. Conclusions

The different approaches to FE simulation were proposed to evaluate the effectiveness of convergence solutions on the corresponding behaviour of a re-entrant cell, such as in polymeric auxetic materials. Regarding DOF, CPU time, and number of elements, the results show that the second-order P-method was the most efficient solution for a re-entrant cell subjected to axial loading. Moreover, there was a good agreement between the converged numerical results (H- and P-methods) and the analytical solution. This research presents that the P-method is the most reliable approach to simulate the re-entrant cell. During the second section of this study, the re-entrant cell was used for generating an auxetic cellular tube. The convergence study was repeated for a cellular tube and the results showed that the second-order P-version formulation was the more efficient method to model re-entrant structures. Polyurethane foams are one of the most common auxetic structures in the field of energy absorption enhancement. The cellular tube was subjected to tensional and compressive loading, and the module of elasticity and Poisson’s ration for different aspect ratios were calculated. A nonlinear analysis was performed to compare the dynamic response of a cellular tube and solid tube. The crashworthiness indicators were addressed and the results were compared with equivalent solid tubes. The results show that the auxetic cellular tubes had better responses against compressive loading.

## Figures and Tables

**Table 1:** Nomenclatures used in the present study.

Symbol	Unit	Description
$L_{1}$	mm	Cellular wall length 1
$L_{2}$	mm	Cellular wall length 2
A	mm^2^	Cross-section area for beam component
$F_{1,}F_{2,}$	N	Applied force to the cell
$\theta_{1}$	°	The angle between $L_{1}$ and $L_{2}$
$U_{t}$	J	Total elastic strain energy
V	mm^3^	Unit cell volume
E	GPa	Young’s modulus of the beam
I	Kg·mm^2^	Secondary moment of inertia
P	N	applied axial force
$P_{q}$	N	Virtual applied axial force
$m_{q}$	N·mm	Virtual bending moment

**Table 2:** Mechanical properties of aluminium [34].

Property	Density	Poisson’s Ratio	Elastic Modulus	Yield Stress	Ultimate Stress	Strain Hardening Coefficient	Failure Strain
Unit	(kg/m^3^)	---	(GPa)	(MPa)	(MPa)	---	(%)
Symbol	ρ	υ	*E*	*σ_y_*	*σ_U_*	*n*	$\varepsilon_{f}$
Value	2700	0.3	56	60	215	0.26	36

**Table 3:** Comparison between two numerical approaches, H and P methods, regarding the degree of freedom, strain energy and CPU time for a single unit cell.

	H-Method				P Method		
	Mesh 10 mm	Mesh 5 mm	Mesh 3 mm	Mesh 2 mm	1st Order	2nd Order	3rd Order
DOF	300	588	996	1452	404	612	612
Strain Energy [kJ]	1.67	1.685	1.701	1.702	1.6	1.701	1.702
CPU time [s]	0.8	1.00	1.5	1.7	0.7	0.8	1
Von Mises stress [MPa]	23.2	24.54	25.01	25.02	24.3	25.02	25.02

**Table 4:** Comparison between two numerical approaches: H- and P- methods regarding the degree of freedom, strain energy and CPU time for cellular tubes while L/D = 1.

	H-Method			P Method	
	Mesh 5 mm	Mesh 3 mm	Mesh 2 mm	2nd Order	3rd Order
DOF	588	996	1452	22,562	22,562
Strain Energy (kJ)	376.89	386.75	387.15	387.12	387.12
CPU time (s)	426	570	696	418	447
Von Mises stress (MPa)	36.02	37.84	37.86	37.89	37.9

**Table 5:** FE results for crashworthiness indicators.

Samples	P Max (N)		Pave (N)		CFE (%)		EA (J)		SEA (J/kg)	
	Solid Tube	Cellular Tube	Solid Tube	Cellular Tube	Solid Tube	Cellular Tube	Solid Tube	Cellular Tube	Solid Tube	Cellular Tube
L/D = 1	842	496	271	435	32.07	87.8	627	975	1081	1681
L/D = 2	1040	575	268	429	25	81.4	1422	2334	1225	2012
L/D = 3	1215	513	302	430.1	24.8	83.8	2359	3361	1355	1931
L/D = 4	1362	680	421	529	30.9	77.7	4552	5723	1962	2466
L/D = 5	1752	682	375	563	21.4	82.5	4994	7506	1722	2588

## References

[1] Prawoto Y. (2012). Seeing auxetic materials from the mechanics point of view: A structural review on the negative Poisson’s ratio. Comput. Mater. Sci..

[2] Mohsenizadeh S., Alipour R., Shokri Rad M., Nejad A.F., Ahmad Z. (2015). Crashworthiness assessment of auxetic foam-filled tube under quasi-static axial loading. Mater. Des..

[3] Zhang X., Yang D. (2016). Mechanical properties of auxetic cellular material consisting of re-entrant hexagonal honeycombs. Materials.

[4] Yang W., Li Z.-M., Shi W., Xie B., Yang M.-B. (2004). Review on auxetic materials. J. Mater. Sci..

[5] Rad M.S., Hatami H., Alipouri R., Nejad A.F., Omidinasab F. (2019). Determination of energy absorption in different cellular auxetic structures. Mech. Ind..

[6] Chan N., Evans K.E. (1997). Fabrication methods for auxetic foams. J. Mater. Sci..

[7] Wojciechowski K.W. (2003). Non-chiral, molecular model of negative Poisson ratio in two dimensions. J. Phys. A.

[8] Tretiakov K., Krzysztof V., Wojciechowski W. (2007). Poisson’s ratio of simple planar ‘isotropic’ solids in two dimensions. Physica Status Solidi.

[9] Rad M.S., Prawoto Y., Ahmad Z. (2014). Analytical solution and finite element approach to the 3D re-entrant structures of auxetic materials. Mech. Mater..

[10] Imbalzano G., Tran P., Ngo T., Lee P.V.S. (2017). Three-dimensional modelling of auxetic sandwich panels for localised impact resistance. J. Sandw. Struct. Mater..

[11] Rueger Z., Roderic S. (2016). Lakes, Cosserat elasticity of negative Poisson’s ratio foam: Experiment. Smart Mater. Struct..

[12] Koumlis S., Lamberson L. (2019). Strain Rate Dependent Compressive Response of Open Cell Polyurethane Foam. Exp. Mech..

[13] Yao Y., Yun L., Xu Y., Wang B., Li J., Deng L., Lu H. (2018). Fabrication and characterization of auxetic shape memory composite foams. Compos. Part B.

[14] Mohsenizadeh S., Alipour R., Nejad A.F., Rad M.S., Ahmad Z. (2015). Experimental investigation on energy absorption of auxetic foam-filled thin-walled square tubes under quasi-static loading. Procedia Manuf..

[15] Liu Y., Hong H. (2010). A review on auxetic structures and polymeric materials. Sci. Res. Essays.

[16] Rad M.S., Ahmad Z., Alias A. (2015). Computational approach in formulating mechanical characteristics of 3D star honeycomb auxetic structure. Adv. Mater. Sci. Eng..

[17] Grima J.N., Elaine M., Daphne A. (2011). Auxetic behaviour from connected different-sized squares and rectangles. Proc. R. Soc. A.

[18] Chetcuti E., Ellul B., Manicaro E., Brincat J.-P., Attard D., Gatt R., Grima J.N. (2014). Modeling auxetic foams through semi-rigid rotating triangles. Physica Status Solidi.

[19] Grima J.N., Gatt R., Alderson A., Evans K.E. (2005). On the potential of connected stars as auxetic systems. Mol. Simul..

[20] Gibson Lorna J., Michael F.A. (1999). Cellular Solids: Structure and Properties.

[21] Masters I.G., Evans K.E. (1996). Models for the elastic deformation of honeycombs. Compos. Struct..

[22] Rahmandoust M., Andreas Ö. (2012). On finite element modeling of single-and multi-walled carbon nanotubes. J. Nanosci. Nanotechnol..

[23] Zhang X.C., Ding H.M., An L.Q., Wang X.L. (2015). Numerical investigation on dynamic crushing behavior of auxetic honeycombs with various cell-wall angles. Adv. Mech. Eng..

[24] Hu L., Fanfan Y., Tongxi Y. (2013). Effect of cell-wall angle on the in-plane crushing behaviour of hexagonal honeycombs. Mater. Des..

[25] Zou Z., Reid S.R., Tan P.J., Li S., Harrigan J.J. (2009). Dynamic crushing of honeycombs and features of shock fronts. Int. J. Impact Eng..

[26] Hu L.L., Yu T.X. (2010). Dynamic crushing strength of hexagonal honeycombs. Int. J. Impact Eng..

[27] Hu L.L., Yu T.X. (2013). Mechanical behavior of hexagonal honeycombs under low-velocity impact–theory and simulations. Int. J. Solids Struct..

[28] Lu Z.-X., Qiang L., Zhen-Yu Y. (2011). Predictions of Young’s modulus and negative Poisson’s ratio of auxetic foams. Physica Status Solidi.

[29] Wang H., Lu Z., Yang Z., Li X. (2019). A novel re-entrant auxetic honeycomb with enhanced in-plane impact resistance. Compos. Struct..

[30] Zhao X., Gao Q., Wang L., Yu Q., Ma Z.D. (2018). Dynamic crushing of double-arrowed auxetic structure under impact loading. Mater. Des..

[31] Hu L.L., Zhou M.Z., Deng H. (2019). Dynamic indentation of auxetic and non-auxetic honeycombs under large deformation. Compos. Struct..

[32] Reid S.R., Peng C. (1997). Dynamic uniaxial crushing of wood. Int. J. Impact Eng..

[33] Ruan D., Lu G., Wang B., Yu T.X. (2003). In-plane dynamic crushing of honeycombs—A finite element study. Int. J. Impact Eng..

[34] Peirovi S., Pourasghar M., Nejad A.F., Hassan M.A. (2017). A study on the different finite element approaches for laser cutting of aluminum alloy sheet. Int. J. Adv. Manuf. Technol..

[35] Taghipour A., Parvizian J., Heinze S., Düster A. (2016). p-version finite elements and finite cells for finite strain elastoplastic problems. PAMM.

[36] Zeng J., Hu H. (2018). Finite Element Analysis of Three-Dimensional (3D) Auxetic Textile Composite under Compression. Polymers.

[37] Dobnik Dubrovski P., Novak N., Borovinšek M., Vesenjak M., Ren Z. (2019). In-Plane Behavior of Auxetic Non-Woven Fabric Based on Rotating Square Unit Geometry under Tensile Load. Polymers.

[38] Zhang W., Zhao S., Sun R., Scarpa F., Wang J. (2019). In-Plane Mechanical Behavior of a New Star-Re-Entrant Hierarchical Metamaterial. Polymers.

[39] Abaqus (2014). Version. 6.14 Documentation.

[40] Marks L.W., Gardner T.N. (1993). The use of strain energy as a convergence criterion in the finite element modelling of bone and the effect of model geometry on stress convergence. J. Biomed. Eng..

[41] Lin C.-L., Chih-Han C., Chia-Shin C., Chau-Hsiang W., Huey-Er L. (1999). Automatic finite element mesh generation for maxillary second premolar. Computer Methods Programs Biomed..

[42] Lim H., Corbett C., Battaile J., Bishop E., James W. (2019). Investigating mesh sensitivity and polycrystalline RVEs in crystal plasticity finite element simulations. Int. J. Plast..

[43] Ng T.P., Koloor S.S.R., Djuansjah J.R.P., Kadir M.A. (2017). Assessment of compressive failure process of cortical bone materials using damage-based model. J. Mech. Behave. Biomed. Mater..

[44] Koloor S.S.R., Rahimian-Koloor S.M., Karimzadeh A., Hamdi M., Petrů M., Tamin M.N. (2019). Nano-level damage characterization of graphene/polymer cohesive interface under tensile separation. Polymers.

[45] Koloor S.S.R., Karimzadeh A., Tamin M.N., Abd Shukor M.H. (2018). Effects of Sample and Indenter Configurations of Nanoindentation Experiment on the Mechanical Behavior and Properties of Ductile Materials. Metals.

